# Childhood-onset granulomatosis with polyangiitis and microscopic polyangiitis: systematic review and meta-analysis

**DOI:** 10.1186/s13023-016-0523-y

**Published:** 2016-10-22

**Authors:** Michele Iudici, Pierre Quartier, Benjamin Terrier, Luc Mouthon, Loïc Guillevin, Xavier Puéchal

**Affiliations:** 1 National Referral Center for Rare Systemic Autoimmune Diseases, Department of Internal Medicine, Hôpital Cochin, Assistance Publique–Hôpitaux de Paris (APHP), Université Paris Descartes, Paris, France; 2 Present Address: Rheumatology Unit, Second University of Naples, Naples, Italy; 3 Pediatric Immunology-Hematology and Rheumatology Unit, Hôpital Necker–Enfants Malades, APHP, IMAGINE Institute and Université Paris Descartes, Paris, France

**Keywords:** Granulomatosis with polyangiitis, Microscopic polyangiitis, Paediatrics

## Abstract

**Background:**

The data from cohorts of childhood-onset granulomatosis with polyangiitis (GPA) and microscopic polyangiitis (MPA) remain scarce and heterogeneous. We aimed to analyse the features at presentation, therapeutic approaches and the disease course of these rare diseases.

**Methods:**

Electronic searches of Medline and the Cochrane Central Register of Controlled trials database were conducted. We also checked the reference lists of the studies included and other systematic reviews, to identify additional reports. We included all cohorts, cross-sectional studies or registries reporting features at presentation or outcomes in patients with a diagnosis of childhood-onset GPA or MPA (age <18 years). The pooled prevalence of clinical manifestations at presentation, ANCA and induction therapies for GPA and MPA was calculated.

**Results:**

We reviewed 570 full texts and identified 14 studies on GPA and 8 on MPA. Childhood-onset GPA and MPA occurred predominantly in female subjects during adolescence. For GPA, ear-nose-throat (ENT) disease (pooled prevalence 82 % [95 % CI 78–87]), constitutional symptoms (73 % [95 % CI 55–88]), renal (65 % [95 % CI 49–79]), and lower respiratory tract (61 % [95 % CI 48–74]) manifestations were the most frequently reported at presentation. Renal disease was a hallmark of MPA (94 % [95 % CI 89–97]). ANCA were detected in >90 % of children with GPA or MPA. Combined corticosteroids and cyclophosphamide was the most frequently used first remission-inducing treatment for GPA (76 % [95 % CI 69–82]) and MPA (62 % [95 % CI 20–96]). Relapses occurred more frequently in GPA (67–100 %) than in MPA (25–50 %). The leading causes of death were the disease itself, and infections.

**Conclusions:**

Childhood-onset MPA and GPA remain severe diseases with frequent relapses and a high cumulative morbidity. Survival and disease-free survival need to be improved.

**Electronic supplementary material:**

The online version of this article (doi:10.1186/s13023-016-0523-y) contains supplementary material, which is available to authorized users.

## Background

Systemic vasculitis in children accounts for 2 to 10 % of the conditions evaluated in paediatric rheumatology clinics [1, 2]. IgA vasculitis and Kawasaki disease are the most common [3], whereas childhood-onset anti-neutrophil cytoplasmic antibody (ANCA)-associated vasculitis (AAV), including granulomatosis with polyangiitis (GPA, Wegener’s), eosinophilic granulomatosis with polyangiitis (EGPA, Churg-Strauss) and microscopic polyangiitis (MPA) are more rare. Our knowledge of these diseases is based mostly on small cohort studies or, more frequently, case series or reports. Since the development of the first specific paediatric classification of vasculitis by the European League Against Rheumatism (EULAR) and the Paediatric Rheumatology European Society (PReS) in 2006 [4], the number of cohorts of patients with paediatric AAV, mostly GPA, reported has steadily increased [5–12].

Nevertheless, the data from such cohorts remain scarce and heterogeneous, and no attempt has yet been made to outline the main features of these rare diseases more precisely.

We conducted a systematic literature review and meta-analysis: 1) to summarize the principal clinical and demographic features on presentation; 2) to describe the course of the disease and 3) to describe the therapeutic approaches reported for children with GPA and MPA.

## Methods

### Literature search

We performed a systematic literature review and a meta-analysis, to obtain a best estimate of the prevalence of clinical manifestations at presentation and to review the treatments given to patients with childhood-onset GPA and MPA. The Meta-analyses of Observational Studies in Epidemiology (MOOSE) guidelines were followed [13]. We conducted a literature search on Medline and the Cochrane Central Register of Controlled trials database from January 1, 1950 to December 31, 2015, as reported on Additional file 1. We also checked the reference lists of the studies included and other systematic reviews on the topic, to identify additional reports.

### Inclusion criteria and procedure

We included all cohorts, cross-sectional studies or registries reporting data for features at presentation or outcomes in patients with a diagnosis of childhood-onset GPA or MPA (age <18 years). All papers providing a baseline description of the study population were considered. Studies reporting only data for specific subgroups of patients (e.g. only patients with nephritis) were excluded.

The titles and abstracts of all the references identified were reviewed independently by two of the authors (MI, XP). The full text of the articles considered potentially relevant was then screened and checked for eligibility. Any disagreements about article inclusion were resolved at this stage.

### Data extraction

We used standardized data extraction forms. We recorded the clinical manifestations at diagnosis, and the number of patients with at least one clinical manifestation within each organ/system domain, if specified. If the authors reported only the number of patients with a particular single clinical manifestation but not the sum of patients with at least one manifestation within a domain (e.g. the number of patients with lung nodules etc., but not the total number of patients with respiratory involvement), we used the lowest estimate for the pooling of prevalence data. Clinical manifestations that were not described were considered to be absent.

Remission and relapses were defined according to EULAR recommendations [14]. We checked the accuracy of data extraction, and any inconsistencies were discussed and resolved.

### Quality scoring

Two authors independently rated each paper for the risk of bias, based on the Methodological Evaluation of Observational research checklist, which was adapted for the specific research question [15] (Additional file 2).

### Statistical analyses

All point estimates of analyses and their 95 % confidence intervals (95 % CI) were calculated by inverse variance weighting. Heterogeneity was assessed by carrying out χ^2^ tests on Cochran’s Q statistic and by calculating I^2^. If heterogeneity was high (I^2^ > 50 %), we used random-effect models. Forest plots were constructed. We considered *p*-values <0.05 to be statistically significant. Data were analysed with MetaXL (MetaXL 1.3, EpiGear International Brisbane). We did not use funnel plots to assess publication bias because this technique was not applicable [16].

## Results

### Literature search

The literature search identified 570 hits in PubMed, and 0 in the Cochrane Library (Additional file 3). Thirty-three articles were considered to be potentially relevant. Another 12 potentially relevant articles were identified by an additional search and by screening the references of the included articles. Overall, we included 14 studies on GPA and eight on MPA.

## Granulomatosis with polyangiitis

### Characteristics of the studies included

We analysed data from 13 retrospective studies [5–7, 9–12, 17–22] and one prospective cohort [23]. One study included two 20-year-old patients [17] and another used an upper limit of 19 years for the definition of childhood-onset AAV [23], but we decided to retain these studies in the analysis to prevent the loss of useful information. In total, data for 294 patients (69 % girls) were included. The main characteristics of studies included are summarised in Table 1.

**Table 1:** Main characteristics of the studies on childhood-onset granulomatosis with polyangiitis and features of the patients at presentation

First author	Stegmayr [20]	Cabral [6]	Rottem [24]	Akikusa [5]	Tahghighi [22]	Gajic-Veljic [23]	Iudici [11]	Sacri [12]	Arulkumaran [7]	Wong [19]	Orlowski [18]	Bohm [9]	Kosalka [10]
Speciality	Paediatric Nephrology	Paediatric Rheumatology	Paediatrics	Paediatric Rheumatology	Paediatric Rheumatology	Paediatric Dermatology	Adult Internal Medicine	Paediatric Nephrology and Rheumatology	Adult Rheumatology	Paediatrics	Paediatrics	Paediatrics	Paediatrics
Country	Sweden, Germany	USA, Canada	USA	Canada	Iran	Serbia	France	France	UK	UK	USA	International	Poland
Year of publication	2000	2009	1993	2007	2013	2013	2015	2015	2011	1998	1978	2014	2014
Years of enrolment	NA	since 2004	NA	1984–2005	2002–2011	1992–2011	1965–2014	1986–2011	1996–2010	1986–1991	1952–1976	2000–2010	1995–2013
Study design	Retrospective	Cross-sectional 2004–2007: retrospectively collected March 2007-November 2008: prospectively collected	Prospective	Retrospective	Cross-sectional Retrospective	Retrospective	Retrospective	Retrospective	Retrospective	Retrospective	Retrospective	Retrospective	Retrospective
Number of patients	7	65	23	25	11	3	25	28	7	12	6	56	9
Ethnicity (*n*)	Caucasian	41 Caucasian, 4 mixed, 3 East Indian, 3 African American, 2 Hispanic, 1 Asian, 1 Middle Eastern, 6 not available	NA	White 21/25	NA	NA	19 Caucasian	21 Caucasian	5 Caucasian, 2 Afro-Carribean	NA	6 Caucasians	46 Caucasian	NA
F (*n*)	3	43	16	20	5	3	18	21	5	8	3	38	6
Median age at onset (range), years	NA	NA	15.4 (9.3–19.4)	NA	NA	11 (6–16)	NA	NA	11.5 (9–15)	9 (0.5–14)	17 (13–20)	11.7 (1st and 3rd quartile 8.5–14.5)	12 (88–16)
Median age at diagnosis (range), years	14 (11–18)	14.2 (4–17)		14.5 (8.7–17.1)	11 (6–15)		14 (2–17)	12.8 (10.1–14.6)					
Classification/diagnostic tool used	Presence of kidney biopsy, upper and lower respiratory tract disease, ANCA+	MD diagnosis	Clinical history compatible with GPA and histopathological evidence of vasculitis or granulomas, or both	ACR Criteria	EULAR/PRINTO/PRES	Clinical history compatible with GPA and histo-pathological evidence of vasculitis or granulomas, or both	EULAR/PRINTO/PRES and Revised Chapel-Hill	EULAR/PRINTO/PRES	EULAR/PRINTO/PRES	ACR Criteria	Histological examination in all patients	EULAR/PRINTO/PRES	EULAR/PRINTO/PRES or ACR criteria
cANCA positivity (ELISA), *n* (%)	6/7 (85.7)	43 (66.2)	NA	13/15 (86.7)	11 (100)	2 (66.6)	10/18 (55.5)	18/28 (64.2)	NA	4 (33.3)	NA	34/51 (66.6)	NA
pANCA positivity (ELISA), *n* (%)	1/7 (14.2)	8 (12.3)	NA	NA	2 (18)	1 (33.3)	4/18 (22.2)	6/28 (21.4)	NA	6 (50.0)	NA	13/50 (26.0)	NA
cANCA positivity (IFI), *n* (%)	NA	43 (66.2)	NA	13/15 (86.7)	11 (100)	2 (66.6)	12/20 (60.0)	NA	4/7 (57.1)	6 (50.0)	NA	NA	NA
pANCA positivity (IFI), *n* (%)	NA	14 (21.5)	NA	2/15 (13.3)	NA	1 (33.3)	4/20 (20.0)	NA	1/7 (14.2)	1 (14.2)	NA	NA	NA
Clinical manifestations													
Systemic, *n* (%)	7 (100)	58 (89.2)	5 (71.4)	24 (96.0)	9 (81.8)	3 (100)	17 (68.0)	23 (82.1)	3 (42.8)	0	5 (83.3)	50 (89.2)	8 (88.8)
Mucocutaneous, *n* (%)	2 (28.5)	23 (35.4)	2 (8.6)	8 (32.0)	3 (27.2)	3 (100)	6 (24.0)	15 (53.5)	4 (57.1)	10 (83.3)	3 (50.0)	36 (64.2)	4 (44.4)
Musculoskeletal, *n* (%)	4 (57.1)	37 (56.9)	7 (30.4)	24 (96.0)	3 (27.2)	2 (66.6)	9 (36.0)	16 (57.1)	5 (71.4)	9 (75.0)	2 (33.3)	33 (58.9)	3 (33.3)
Ocular, *n* (%)	0	24 (36.9)	3 (13.0)	13 (52.0)	2 (18.8)	2 (66.6)	7 (28.0)	6 (21.4)	3 (42.8)	0	1 (16.6)	19 (33.9)	1 (11.1)
Ear, nose, and throat, *n* (%)	7 (100)	52 (80.0)	20 (87)	21 (84.0)	8 (72.7)	2 (33.3)	21 (84.0)	21 (75.0)	4 (57.1)	11 (91.6)	6 (100)	51 (91.0)	5 (55.5)
Respiratory, *n* (%)	6 (85.7)	52 (80.0)	5 (21.7)	20 (80.0)	2 (18.1)	0	17 (68.0)	19 (67.8)	3 (42.8)	7 (58.3)	5 (83.3)	44 (78.5)	7 (77.7)
Cardiovascular, *n* (%)	0	0	2 (8.7)	5 (20.0)	1 (9.0)	0	0	0	0	0	0	0	0
Gastrointestinal, *n* (%)	0	27 (41.5)	0	3 (12.0)	3 (27.2)	0	5 (20.0)	5 (17.8)	1 (14.2)	6 (50.0)	0	9 (16.0)	5 (55.5)
Neurological, *n* (%)	0	16 (24.6)	1 (4.3)	2 (8.0)	1 (9.0)	0	1 (4.0)	1 (3.5)	1 (14.2)	2 (16.6)	1 (16.6)	8 (14.2)	1 (11.1)
Renal, *n* (%)	7 (100)	49 (75.4)	2 (8.6)	22 (88.0)	4 (36.3)	3 (100)	9 (36.0)	22 (78.5)	4 (57.1)	4 (33.3)	5 (83.3)	38 (67.8)	8 (88.8)
Treatment													
Oral GCs ± IS, *n* (%)	7 (100)	60 (92.3)	23 (100)	25 (100)	NA	3 (100)	21 (84.0)	28 (100)	NA	NA	5 (83.3)	NA	9 (100)
GCs ± CYC, *n* (%)	6 (85.7)	54 (83.0)	18 (78.2)	15 (60.0)	NA	3 (100)	18 (72.0)	NA	NA	NA	3 (50.0)	NA	9 (100)
GCs ± MTX, *n* (%)	0	7 (10.7)	1 (4.3)	5 (20.0)	NA	0	2 (8.0)	NA	NA	NA	0	NA	0
GCs ± AZA, *n* (%)	0	0	2 (8.6)	0	NA	0	2 (8.0)	NA	NA	NA	0	NA	0
Plasmapheresis, *n* (%)	4 (57.1)	9 (13.8)	0	0	NA	1 (33.3)	1 (4.0)	NA	NA	NA	0	NA	0

*NA* not available, *ELISA* enzyme-linked immunosorbent assay, *IFI* indirect immunofluorence, *GCs* glucocorticoids, *IS* immunosoppressors, *CYC* cyclophosphamide, *MTX* methotrexate, *AZA* azathioprine, *GPA* granulomatosis with polyangiitis

### Risk of bias

The most frequent sources of bias were the sampling framework and the case definition for GPA, followed by patient selection (Additional file 2: Tables S1 and S2).

### Clinical and laboratory features on entry into the study

Thirteen of the 14 studies included assessed the features of the patients on entry into the study [5–7, 9–12, 17–19, 21–23]. These studies included 277 patients in total: 145/194 (75 %) were Caucasian (data available for seven studies), with a median age at disease onset of 11.6 years (data available for seven studies) and a median age at diagnosis of 14 years (range: 4–17) (data available for six studies). Prevalence for the involvement of each organ/system is shown in Table 2.

**Table 2:** Prevalence for the involvement of each organ/system at first consultation in childhood-onset granulomatosis with polyangiitis and microscopic polyangiitis

Organ/system	ENT	Systemic	Renal	Lower respiratory tract	Musculoskeletal	Cutaneous	Ocular	Gastrointestinal	Neurological	Cardiovascular
GPA										
Pooled prevalence	82	73	65	61	55	44	24	19	13	4
95 % CI	78–87	55–88	49–79	48–74	43–67	32–57	15–34	10–30	9–17	1–9
I^2^	26	88	84	77	72	75	64	72	15	61
MPA										
Pooled prevalence	3	79	94	37	57	44	7	28	18	2
95 % CI	1–7	65–90	89–97	24–52	27–86	27–61	3–11	17–41	7–34	0–5
I^2^	0	64	47	67	89	73	0	55	72	0

**Fig. 1:**
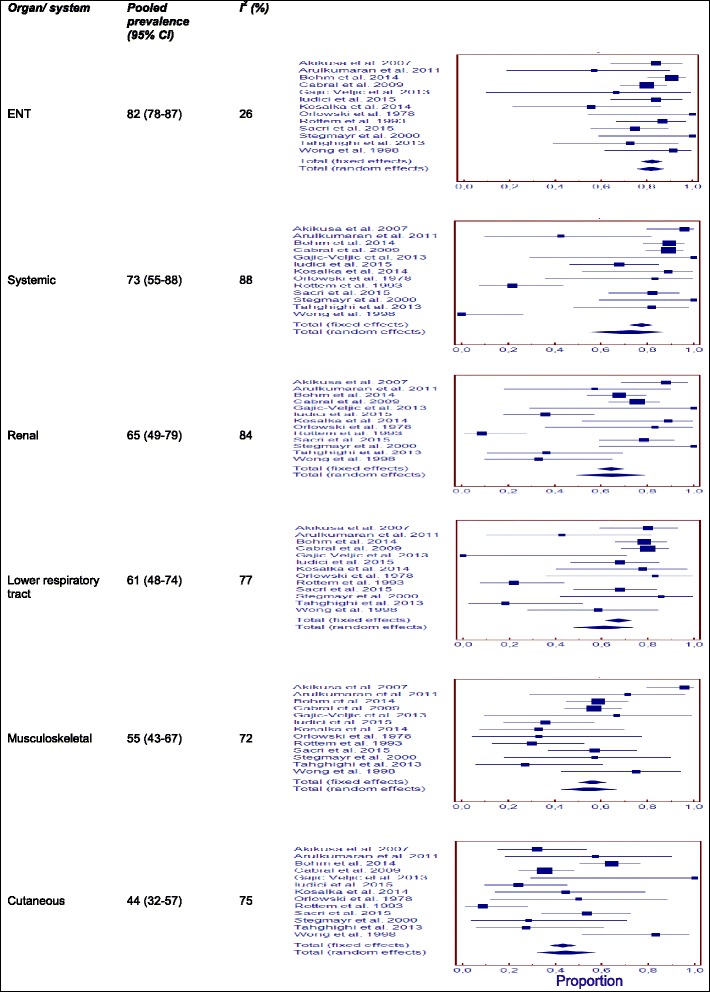
Pooled prevalence for the most frequent organ/system involvement at first consultation in childhood-onset granulomatosis with polyangiitis

ENT system involvement was the most frequent manifestation observed, followed by constitutional symptoms, renal, lower respiratory tract, musculoskeletal and cutaneous involvement (Fig. 1). Mild heterogeneity was observed only for ENT and neurologic involvement.

None of the patients had baseline gangrene or retinal vasculitis, two of the “major” clinical manifestations according to the modified Birmingham Vasculitis Activity Score [24]. CNS (central nervous system) involvement occurred in 3 % (95 % CI 1–7 %; I^2^ = 31 %) of patients, but no detailed information was provided. Other major manifestations included scleritis/episcleritis in 2 % (95 % CI 1–4 %; I^2^ = 43 %), ischemic abdominal pain in 2 % (95 % CI 1–4 %; I^2^ = 64 %), haemoptysis/alveolar haemorrhage in 16 % (95 % CI 6–29 %; I^2^ = 84 %), and renal problems requiring dialysis in 2 % (95 % CI 1–5 %; I^2^ = 48 %) of patients. Subglottic stenosis and saddle nose were observed at presentation in 9 % (95 % CI 6–12 %; I^2^ = 0 %) and 5 % (95 % CI 1–11 %; I^2^ = 64 %) of patients, respectively, and lung nodules were observed in 10 % of patients (95 % CI 2–22 %; I^2^ = 84 %).

A sensitivity analysis was performed. We first excluded the two studies including adult patients [7, 11], with no major impact on prevalence estimates and then the nephrology surveys [12, 19], resulting in a lower prevalence of renal disease at admission (Additional file 4: Table S5). Following the grouping of studies by geographic origin (Europe or North America), we found that the prevalence of skin manifestations was higher in Europe (prevalence 55 % [95 % CI 32–76] vs. 28 % [95 % CI 14–45 %]; *p* = 0.01) [7, 10–12, 18, 19, 22], whereas the prevalence of neurological involvement was higher in studies from North America [5, 6, 17, 23] (17 % [95 % CI 10–24] vs. 7 % [95 % CI 2–14]; *p* = 0.043).

Seven studies provided information about ANCA detection by immunofluorescence methods [5–7, 11, 17, 21, 22]. Positive results were obtained for c-ANCA in 71 % (95 % CI 56–84, I^2^ = 59 %), and for p-ANCA in 20 % (95 % CI 13–27, I^2^ = 0 %) of patients. The estimated prevalence of anti-PR3 (proteinase 3) antibodies was 69 % (95 % CI 58–80, I^2^ = 60 %), whereas that of anti-MPO (myeloperoxidase) antibodies was 21 % (95 % CI 16–27, I^2^ = 25 %) (data available from 9 studies) [5, 6, 9, 11, 12, 18, 19, 21, 22].

### Induction therapies and drug-related adverse events

The drugs used for induction therapy were reported in nine studies [5, 6, 10–12, 17, 19, 22, 23]. Corticosteroids were the most prescribed drugs (prevalence 95 %; 95 % CI 92–98; I^2^ = 35 %), but it was not possible to determine the dose most frequently used. Immunosuppressants were also used (in monotherapy or with corticosteroids): cyclophosphamide in 76 % (95 % CI 69–82; I^2^ = 27 %) of patients, methotrexate in 10 % (95 % CI 6–16; I^2^ = 0 %), azathioprine in 2 % (95 % CI 0–5; I^2^ = 26 %) and plasmapheresis in 8 % of patients (95 % CI 1–21; I^2^ = 72 %). With the exception of plasmapheresis, immunosuppression protocols were very similar between studies. The other drugs used for induction therapy included immunoglobulins, cyclosporine, colchicine and rituximab.

The principal drug-related adverse events reported were infections. Infertility, hemorragic cystitis, cataracts, glaucoma, osteoporosis, steroid myopathy, Cushing syndrome, and depression were also reported. Growth retardation was observed in two studies [5, 20], but not confirmed in a third one [11]. Only one case of breast cancer diagnosed at age of 30 was reported.

### Follow-up data for cumulative clinical manifestations, relapse, and survival

At least one item of information about the course of the disease was reported in 11 articles [5, 7, 10–12, 17, 19–23]. The duration of the observation period was reported in four studies, and ranged from 2.7 to 18.5 years [5, 7, 11, 23]. At least one relapse occurred in 67 to 100 % of patients. Survival data were available for 161 patients: 14 of these patients died (at a mean age (±SD) of 18 ± 11 years; based on data for eight patients), from sepsis in two patients, fungal infection in one, pulmonary haemorrhage in three, chronic lung disease due to GPA in two, multiple-organ failure in one, and a heroin overdose in one. Cumulative incidence was 91 to 100 % for ENT involvement, up to 48 % for subglottic stenosis, up to 60 % for ocular disease and up to 88 % for renal involvement.

## Microscopic polyangiitis

### Literature search and characteristics of the studies included

We analysed data from eight retrospective cohorts [11, 12, 25–30] for a total of 130 patients (86/109 patients were female [79 %]). The main organ/system involvement are summarised in Table 2 and the main characteristics of studies included in Table 3.

**Table 3:** Main characteristics of the studies on childhood-onset microscopic polyangiitis and features of the patients at presentation

First author	Iudici [11]	Sacri [12]	Bakkaloglu [25]	Peco-Antic [27]	Basu [30]	Sun [29]	Hattori [26]	Yu [28]
Speciality	Adult Internal Medicine	Paediatric Nephrology and Rheumatology	Paediatric Nephrology and Rheumatology	Paediatric Nephrolology	Paediatric Nephrolology	Paediatric Nephrology	Paediatric Nephrology	Paediatric Nephrology
Year of publication	2015	2015	2001	2006	2015	2014	2001	2006
Years of enrolment	1965–2014	1986–2011	1990–1999	1998–2003	2011–2014	2003–2013	1990–1997	1998–2004
Study design	Retrospective	Retrospective	Retrospective	Retrospective	Retrospective	Retrospective	Retrospective	Retrospective
Number of patients	4	38	10	7	11	20	21	19
Ethnicity	3 Caucasian, 1 Asian	26 (68 %) Caucasian	NA	NA	NA	NA	NA	NA
Female, number (%)	0	34 (89)	6 (60)	6 (86)	6 (54)	16 (80)	NA	18 (95)
Median age at onset	NA	NA	NA	12 (7–15)	7.6 (4.3–11.8)	10 (1.9–16.8)	NA	10.8 ± 2.8
Median age at diagnosis	13 (3–16)	11.2 (8.9–12.3)	12 (8–17)	NA	NA	NA	NA	NA
Classification/diagnostic tool used	Revised Chapel-Hill	According to Watts et al. [40]	Typical biopsy findings for renal or non-renal tissues	Inclusion criteria: 1) symptoms suggestive of MPA; 2) Biopsy-proven GN; 3) MPO-ANCA+	Revised Chapel-Hill CC	Revised Chapel-Hill CC	Chapel-Hill	Chapel-Hill
cANCA positivity (ELISA)	1/1	4/38	0	NA	0	2/17	NA	0
pANCA positivity (ELISA)	0/1	33/38	10 (100)	7 (100)	11	16/17	NA	19/19
cANCA positivity (IFI)	1/2	0	0	NA	0	2/20	NA	0
pANCA positivity (IFI)	1/2	NA	9 (90)	NA	10/10	18/20	NA	19/19
Clinical manifestations								
Systemic	4 (100.0)	29 (78.0)	10 (100.0)	7 (100.0)	8 (72.7)	10 (50.0)	18 (85.7)	12 (63.1)
Mucocutaneous	2 (50.0)	13 (34.2)	7 (70.0)	7 (100.0)	3 (27.2)	3 (15.0)	8 (38.0)	6 (31.5)
Musculoskeletal	2 (50.0)	11 (28.9)	6/9 (66.7)	7 (100.0)	11 (100.0)	2 (10.0)	7 (33.0)	0
Ocular	0	3 (7.8)	0	0	0	1 (5.0)	2 (9.5)	1 (5.2)
Ear, nose, and throat	0	0	0	0	0	0	2 (9.5)	2 (10.5)
Respiratory	0	11 (28.9)	3 (30.0)	4 (57.1)	5 (45.4)	3 (15.0)	13 (61.9)	10 (52.6)
Cardiovascular	1 (25.0)	0	0	0	1 (9.0)	0	0	0
Gastrointestinal	2 (50.0)	4 (10.5)	2/9 (22.2)	4 (57.1)	2 (18.1)	3 (15.0)	7 (33.3)	9 (47.3)
Neurological	2 (50.0)	2 (5.2)	2/9 (22.2)	6 (85.7)	1 (9.0)	3 (15.0)	1 (4.7)	1 (5.2)
Renal	4 (100.0)	36 (94.7)	7 (70.0)	7 (100)	11 (100.0)	16 (100.0)	21 (100.0)	19 (100.0)

*NA* not available, *ELISA* enzyme-linked immunosorbent assay, *IFI* indirect immunofluorence

### Risk of bias

The most frequent source of bias was the sampling framework for MPA, followed by patient selection and the definition of MPA (Additional file 2: Tables S3 and S4).

### Clinical and laboratory features on entry into the study

For the 130 MPA patients identified, median age was 10.5 years at disease onset (data available for four studies) and 12 years at diagnosis (data available for three studies).

**Fig. 2:**
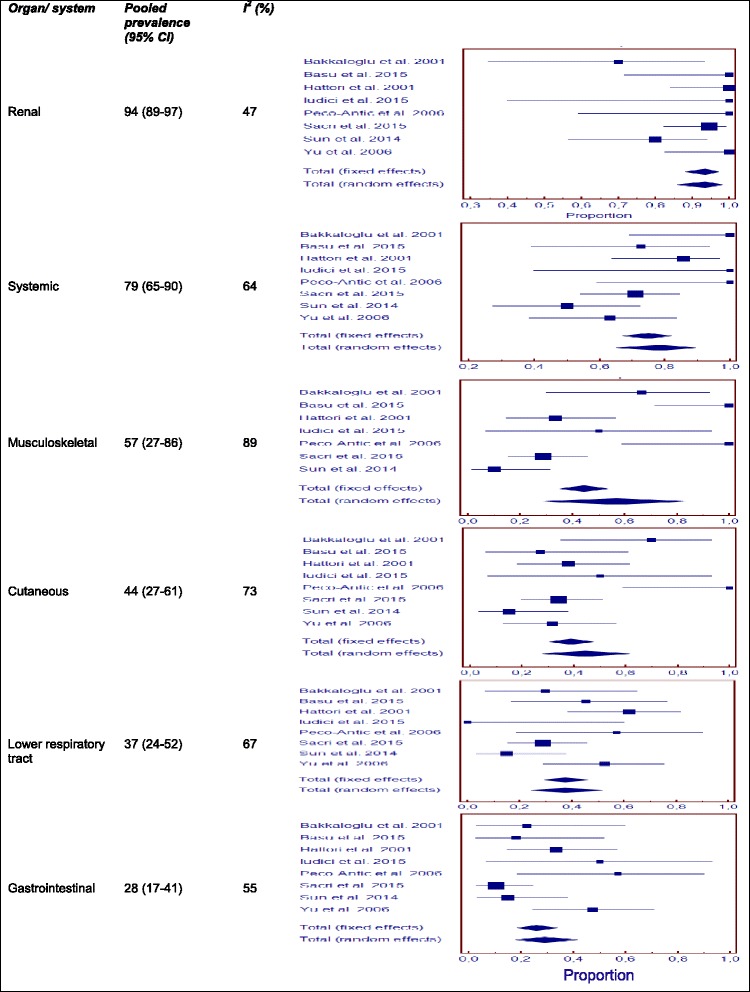
Pooled prevalence for the most frequent organ/system involvement at first consultation in childhood-onset microscopic polyangiitis

Renal disease was the most frequent manifestation on presentation, followed by systemic features, musculoskeletal, cutaneous, lower respiratory tract and gastrointestinal system involvement (Table 2). Heterogeneity between studies was low for renal, ENT, ocular, and cardiovascular involvement (Fig. 2). Lung infiltrates (18 % [95 % CI 6–35]; I^2^ = 77 %) and alveolar haemorrhage (18 %, [95 % CI 5–35]; I^2^ = 77 %) occurred at similar frequencies. ENT involvement was limited to sinusitis. Seizures were the most frequent neurological manifestation (5 % [95 % CI 2–9]; I^2^ = 44 %) and purpura was the most frequent skin lesion (10 % [95 % CI 0–27]; I^2^ = 86 %).

Following the grouping of the studies by geographic region of origin (Europe or Asia), we found that the prevalence of skin manifestations (66 % [95 % CI 28–96] vs. 29 % [95 % CI 19–40]; *p* = 0.02) was higher in studies from Europe [11, 12, 25, 27] (Additional file 4: Table S6).

Information about immunofluorescence findings for ANCA positivity was available in five studies [11, 25, 28–30]. Positive results were obtained for c-ANCA in 4 % of patients (95 % CI 1–11, I^2^ = 50 %), and for p-ANCA in 94 % of patients (95 % CI 86–99, I^2^ = 35 %). The prevalence of anti-MPO antibodies was 93 % (95 % CI 83–99, I^2^ = 52 %), that of anti-PR3 antibodies was 5 % (95 % CI 1–11, I^2^ = 0 %) (data from 8 studies) [11, 12, 25, 27–29].

### Induction therapies and drug-related adverse events

Six studies reported the drugs used to induce remission [11, 25, 27–30]. In five of these studies, oral or intravenous cyclophosphamide was used with corticosteroids (62 %, 95 % CI 20–96, I^2^ = 90 %). In one study [30], only rituximab was used. If we excluded this study from the analysis, the prevalence of cyclophosphamide use increased to 77 % (95 % CI 45–99, I^2^ 82 %). In one study [27], plasmapheresis was also performed. No detailed information about drug-related adverse events during follow-up was provided. One study stated that no cases of impaired fertility or cancer occurred during follow-up [29]. In another, only infections were described [30].

### Follow-up data for cumulative clinical manifestations, relapse, and survival

Follow-up data were reported in seven articles [11, 12, 25, 27–30]. The duration of the observation period was reported in six studies, and ranged from 4 to 55 months. At least one relapse occurred in 25 to 50 % of the patients (total of 63 patients from four studies) [11, 12, 25, 30]. Twenty-nine patients (22 %) developed end-stage renal disease or a need for dialysis during follow-up. All studies provided survival data. Six children died: one from the rupture of a hepatic aneurysm, one from pulmonary infection, one from cytomegalovirus infection, and no information was available for the other patients.

## Discussion

We conducted this study to provide a better definition of the principal manifestations of childhood-onset GPA and MPA at presentation and their course over time. This study is the first to use a systematic approach including a meta-analysis to analyse these rare paediatric conditions for which a small number of essentially retrospective studies have been published to date.

We confirmed the female predominance of GPA and MPA in children. About 70 % of the GPA patients and 80 % of the MPA patients were girls. This finding contrasts with those for studies in adults, which have reported an absence of difference in frequency between the sexes or a slightly higher frequency in men for [31–33]. We also confirmed that most of these paediatric cases were diagnosed in adolescence.

As expected, the principal manifestations at disease onset were those involving the respiratory tract in patients with GPA and the kidney in patients with MPA. ENT involvement was rare in MPA, but was the most frequent clinical feature at presentation in GPA patients. It is, therefore, a potentially useful additional sign for distinguishing between these two conditions. Saddle nose was already present at presentation in 5 % of GPA patients, confirming previous observations of a higher rate of this complication in children [20, 23, 32]. Sinusitis was the only ENT feature observed in patients with MPA.

Renal disease was the leading feature of MPA, and was observed at onset in almost all children. However, as most of the surveys included were carried out at nephrology centres, there may have been a referral bias. As for GPA, the data from the different cohorts were highly heterogeneous, but renal impairment was diagnosed in about 65 % of patients at presentation. This observation suggests that childhood GPA, when diagnosed, is a multisystem disease rather than a condition “limited” to the upper airways, as proposed in the past [23, 34]. In addition, renal disease is not very common at presentation in adults (17 % of patients in the study by Hoffman et al. [31]) but it generally occurs during the course of the disease. By contrast, it seems to have a much higher incidence at disease onset in children. Moreover, one fifth of the children with MPA displayed progression to ESRD (end-stage renal disease) or a need for dialysis during follow-up, highlighting the severity of renal disease in these children.

In addition to the principal specific manifestations observed at presentation in GPA and MPA patients, systemic features were also frequent in three-quarters of patients at presentation and half the patients presented musculoskeletal symptoms, as also highlighted by a recently published study by the ARChiVe Investigator Network [35]. Eye problems were rare, but were more frequent in GPA than in MPA, whereas gastrointestinal involvement was more frequent in MPA than in GPA patients. Cardiomyopathy was described in only two patients, both with MPA.

Special attention should be paid to alveolar haemorrhage and subglottic stenosis, which may be life-threatening. Alveolar haemorrhage was not rare at disease presentation in either MPA or GPA, being observed in about 18 % of patients. Subglottic stenosis was observed only in GPA patients. Early reports suggested that the incidence of subglottic stenosis was higher in children with GPA than in adults. Rottem et al. [23] found that about 40 % of patients developed subglottic stenosis during 8 years of follow-up, corresponding to rates five times higher than reported in adult surveys [23]. These data, together with those from a UK cohort [20], led to the inclusion of subglottic stenosis in the paediatric classification criteria [4]. Our results give a more precise estimate of the impact of this complication on the course of paediatric GPA. The available data show that a fairly uniform proportion of paediatric cases (9 %) already display subglottic stenosis at presentation. By contrast, incidence was found to vary considerably over time. Cumulative incidence ranged from 16 % [11] to 40–48 % in studies with similar follow-up periods [8, 20, 23]. Overall, these data confirm the previous finding of a higher risk of subglottic stenosis in children than in adults, in whom a cumulative prevalence of 10 % has been reported [36].

A large proportion of the patients tested detected positive for ANCA. Only one MPA patient with anti-PR3 antibodies was reported. However, one fifth of the GPA patients tested positive for anti-MPO antibodies, and this proportion was similar in all studies. The high prevalence of ANCA in children as compared to adults should be borne in mind when paediatricians are considering a diagnosis of one of these diseases.

A number of different treatment regimens were used, but the drugs used to treat GPA differed little between studies, reflecting a relatively uniform approach to the induction of remission over the long time period covered by this meta-analysis. Most of the information about treatment provided by the available reports concerned “traditional” drugs, with rituximab treatment reported in only one patient. This is consistent with the approval of rituximab registration for induction treatment in adult patients with GPA and MPA by the FDA in 2011 and by the EMA in 2013. Reports pointing out a good response to rituximab are rapidly increasing. Cyclophosphamide was the drug most frequently used to treat patients with MPA, and a protocol based on rituximab and mycophenolate mofetil was considered as a first-line treatment in only one study, on 11 patients [30]. In this study, 90 % of the patients were in complete remission at the last follow-up visit.

The paucity of available information made it impossible to define the course of the disease with any degree of precision. For example, a detailed assessment of disease activity over time is lacking. This could have been favoured by the delay in drawing up a specific index for paediatric conditions [37]. However, the available data suggest that most GPA patients experience at least one relapse, whereas disease flares are observed in less than half of all MPA patients. However, end-stage renal disease was a particular problem in children with MPA. About one fifth of the children with MPA presented ESRD, whereas the proportion of GPA patients presenting this condition was much lower. As in adults [38], the development of new therapeutic strategies should help to improve disease-free survival.

The burden of drug-related adverse effects was well described only in papers on GPA. The most frequent problems encountered were infections, followed by damage due to chronic corticosteroid and cyclophosphamide treatments. The data relating to growth retardation in GPA patients were inconsistent.

Overall, 7 % of the patients for whom follow-up data were available died during follow-up, mostly from disease-related and infectious complications. Respiratory complications were the leading causes of disease-related death in children with GPA. These data confirm that GPA and MPA diseases in children are severe and difficult to manage. Long-term survival remains a matter of concern and should be assessed in future reports.

This study has several limitations. First, determining the prevalence of clinical manifestations at the onset of AAV was not the main objective in most of the primary studies. So despite the fact that we used a broad search strategy, it is likely that we missed some relevant studies reporting these data. The large number of additional articles identified by manual searching is consistent with this hypothesis. Moreover, we cannot exclude the possibility that the descriptions of the features of patients at presentation were not always exhaustive. There was also considerable heterogeneity, which was explored through subgroup analysis, although it should be borne in mind that such analyses are known to be subject to certain limitations. Finally, at least one monogenic disease associated with early-onset polyarteritis nodosa such as ADA2 (Adenosine deaminase 2) deficiency has recently been described [39] and other monogenic disorders corresponding to some cases of childhood-onset AAV with a narrower range of clinical features and a specific drug-response pattern may be discovered in the near future.

## Conclusion

We found that the incidence of childhood GPA and MPA was higher in girls than in boys, with most cases diagnosed during adolescence. Renal disease was the main feature at presentation in patients with MPA, whereas ENT was the predominant feature in patients with GPA. Childhood-onset GPA was associated with a higher prevalence of renal disease and nasal deformities at presentation and a higher overall incidence of subglottic stenosis over time than GPA in adults. Relapses were more frequently observed in patients with GPA than in those with MPA, and the leading causes of death were related to the disease itself or to infections. Further improvements in survival and disease-free survival are required in these severe paediatric diseases.

## Additional files

Additional file 1: Search strategy. (DOCX 14 kb)
Additional file 2: Risk of bias assessment. (DOCX 35 kb)
Additional file 3: Flow-chart of paper selection. (DOC 61 kb)
Additional file 4: Subgroup analysis. (DOCX 21 kb)

## Abbreviations

AAV: ANCA-Associated vasculitis
ADA2: Adenosine deaminase 2
ANCA: Anti-neutrophil cytoplasmic antibody
CNS: Central nervous system
EGPA: Eosinophilic granulomatosis with polyangiitis (Churg-Strauss)
EMA: European medicines agency
ENT: Ear, nose and throat
ESRD: End-stage renal disease
EULAR: European league against rheumatism
FDA: Food and drug administration
GPA: Granulomatosis with polyangiitis (Wegener’s)
MOOSE: Meta-analyses of observational studies in epidemiology
MPA: Microscopic polyangiitis
MPO: Myeloperoxidase
PR3: Proteinase 3
PReS: Paediatric rheumatology european society
