# Supplementary material for: Childhood-onset granulomatosis with polyangiitis and microscopic polyangiitis: systematic review and meta-analysis
Source: Orphanet J Rare Dis. 2016 Oct 22;11:141. doi: 10.1186/s13023-016-0523-y (PMC5075395; doi:10.1186/s13023-016-0523-y)
Supplement: Additional file 1: — Search strategy. (DOCX 14 kb) [file 13023_2016_523_MOESM1_ESM.docx]

**Additional file 1. Search strategy**

**Medline**

("Wegener Granulomatosis" [Mesh] OR "Wegener*" [tiab] OR “granulomatosis*” [tiab] OR “Microscopic Polyangiitis” [Mesh] OR “Anti-Neutrophil Cytoplasmic Antibody-Associated Vasculitis” [Mesh] OR “ANCA*”[tiab] OR “Polyangiitis*” [tiab]) AND (“child*” [tiab] OR “juvenile*” [tiab] OR “pediatric*” [tiab] OR “young*” [tiab] OR “infant*” [tiab]).

**The Cochrane Library**

1. MeSH descriptor Anti-Neutrophil Cytoplasmic Antibody-Associated Vasculitis

2. wegener* granulomatosis*:ti,ab

3. polyangiitis*:ti,ab

4. (#1 OR #2 OR #3)

5. child*:ti,ab

6. juvenile*:ti,ab

7. pediatric*:ti,ab

8. young*:ti,ab

9. infant*: ti,ab

10. (#5 OR #6 OR #7 OR #8 OR #9)

11. (#4 AND #11)
