# Supplementary material for: Childhood-onset granulomatosis with polyangiitis and microscopic polyangiitis: systematic review and meta-analysis
Source: Orphanet J Rare Dis. 2016 Oct 22;11:141. doi: 10.1186/s13023-016-0523-y (PMC5075395; doi:10.1186/s13023-016-0523-y)
Supplement: Additional file 2: — Risk of bias assessment. (DOCX 35 kb) [file 13023_2016_523_MOESM2_ESM.docx]

**Additional file 2. Risk of bias assessment**

The assessment of the *External validity* was based on the following questions:

(1) Was the framework for sampling representative of the target population?

(2) Was an appropriate case definition for GPA/MPA used?

(3) Was some form of random selection used to select the sample?

For each question, three options were possible: ‘+’=low risk of bias, ‘−’=possible risk of bias or ‘?’=risk of bias unclear due to poor reporting. The rating procedure for each study is shown below.

We did not assess *Internal validity* due to the lack of standardised definitions for each of the clinical manifestations assessed. We therefore assigned a “possible risk of bias” to all studies for internal validity.

**Table S1**. Criteria used to assess the risk of bias across studies for childhood-onset granulomatosis with polyangiitis

| **External validity** | **Low risk of bias** | **Possible risk of bias** | **Poor reporting** |
| --- | --- | --- | --- |
| *Sampling framework* | Paediatric surveys | Adult surveys | Not reported |
|  | Inpatients | Patients’ associations |  |
|  | Outpatients |  |  |
|  | Primary care |  |  |
|  | Registry |  |  |
| *Case definition for GPA* | 1990 ACR criteria* [1] EULAR/PRES criteria** [2] | Medical judgement | Not reported |
|  | Chapel-Hill nomenclature° [3] |  |  |
|  | Biopsy plus ANCA + plus typical clinical picture |  |  |
| *Random selection of patients* | Consecutive patients  Total registry | Specific inclusion and exclusion criteria | Not reported |

* Criteria for the Classification of GPA (Wegener's) [1]

Nasal or oral inflammation: development of painful or painless oral ulcers or purulent or bloody nasal discharge

Abnormal chest radiograph: chest radiograph showing the presence of nodules, fixed infiltrates, or cavities

Urinary sediment: microhematuria (>5 red blood cells per high power field) or red cell casts in urine sediment

Granulomatous inflammation on biopsy: histologic changes showing granulomatous inflammation within the wall of an artery or in the perivascular or extravascular area (artery or arteriole)

For purposes of classification, a patient shall be said to have GPA if at least 2 of these 4 criteria are present.

** EULAR/PRINTO/PreS criteria for GPA [2]

1. Histopathology: granulomatous infl ammation within the wall of an artery or in the perivascular or extravascular area

2. Upper airway involvement

Chronic purulent or bloody nasal discharge or recurrent epistaxis/crusts/granulomata

Nasal septum perforation or saddle nose deformity

Chronic or recurrent sinus infl ammation

3. Laryngo-tracheo-bronchial involvement

Subglottic, tracheal or bronchial stenoses

4. Pulmonary involvement

Chest x-ray or CT showing the presence of nodules, cavities or fi xed infiltrates

5. ANCA

ANCA positivity by immunofl uorescence or by ELISA (MPO/p or PR3/c ANCA)

6. Renal involvement

Proteinuria >0.3 g/24 h or >30 mmol/mg of urine albumin/creatinine ratio on a spot morning sample

Haematuria or red blood cell casts: >5 red blood cells/high power fi eld or red blood cells casts in the urinary sediment or ≥2+ on dipstick

Necrotising pauci-immune glomerulonephritis

To be classified at least three of the six following criteria are needed:

- Histopathology
- Upper airway involvement
- Laryngo-tracheo-bronchial stenosis
- Pulmonary involvement
- ANCA positivity
- Renal involvement

° 2012 Revised International Chapel Hill Consensus Conference Nomenclature of Vasculitides [3]

Necrotizing granulomatous inflammation usually involving the upper and lower respiratory tract, and necrotizing vasculitis affecting predominantly small to medium vessels (e.g., capillaries, venules, arterioles, arteries and veins). Necrotizing glomerulonephritis is common.

**Table S2**. Criteria used to assess the risk of bias across studies for childhood-onset microscopic polyangiitis

| **External validity** | **Low risk of bias** | **Possible risk of bias** | **Poor reporting** |
| --- | --- | --- | --- |
| *Sampling framework* | Paediatric surveys | Adult surveys | Not reported |
|  | Inpatients | Patients’ associations |  |
|  | Outpatients |  |  |
|  | Primary care |  |  |
|  | Registry |  |  |
| *Case definition for MPA* | Chapel-Hill Nomenclature* [3] Biopsy plus ANCA + plus typical clinical signs | Medical judgement | Not reported |
| *Random selection of patients* | Consecutive patients  Total registry | Specific inclusion and exclusion criteria | Not reported |

* 2012 Revised International Chapel Hill Consensus Conference Nomenclature of Vasculitides [3]

Necrotizing vasculitis, with few or no immune deposits, predominantly affecting small vessels (i.e., capillaries, venules, or arterioles). Necrotizing arteritis involving small and medium arteries may be present. Necrotizing glomerulonephritis is very common. Pulmonary capillaritis often occurs. Granulomatous inflammation is absent.

**Table S3**. Risk of bias for studies of childhood-onset granulomatosis with polyangiitis

| Author | Year | Study design | Country | Sample  size | **Risk of bias** | | |
| --- | --- | --- | --- | --- | --- | --- | --- |
|  |  |  |  |  | Sampling  framework | Case  definition for  GPA | Selection  of patients |
| Akikusa *et al*. [5] | 2007 | Retrospective | Canada | 25 | + | + | - |
| Arulkumaran *et al*. [7] | 2011 | Retrospective | UK | 7 | - | + | - |
| Belostotsky *et al*. [22] | 2002 | Retrospective | UK | 17 | + | + | + |
| Bohm *et al*. [9] | 2014 | Retrospective | International | 56 | - | + | + |
| Cabral *et al*. [6] | 2009 | Cross-sectional | USA, Canada | 65 | + | + | + |
| Gajic-Veljic *et al*. [24] | 2013 | Retrospective | Serbia | 3 | + | - | + |
| Iudici *et al*. [11] | 2015 | Retrospective | France | 25 | - | + | + |
| Kosalka *et al*. [10] | 2014 | Retrospective | Poland | 9 | + | + | + |
| Orlowski *et al*. [18] | 1978 | Retrospective | USA | 6 | + | - | + |
| Rottem *et al*. [19] | 1993 | Prospective | USA | 23 | + | - | + |
| Sacri *et al*. [12] | 2015 | Retrospective | France | 28 | + | + | + |
| Stegmayr *et al*. [21] | 2000 | Retrospective | Sweden, Germany | 7 | + | + | + |
| Tahghighi *et al.* [23] | 2013 | Retrospective | Iran | 11 | + | + | + |
| Wong *et al*. [20] | 1998 | Retrospective | UK | 12 | + | + | + |

**Legend.** + Low risk of bias; - Possible risk of bias; ? Poor reporting

**Table S4**. Risk of bias in studies of childhood-onset microscopic polyangiitis

| Author | Year | Study design | Country | Sample  size | **Risk of bias** | | |
| --- | --- | --- | --- | --- | --- | --- | --- |
|  |  |  |  |  | Sampling  framework | Case  definition for  MPA | Selection  of patients |
| Bakkaloglu *et al*. [26] | 2001 | Retrospective | Turkey | 10 | - | - | - |
| Basu *et al*. [31] | 2015 | Retrospective | India | 11 | + | + | + |
| Hattori *et al.* [27] | 2001 | Retrospective | Japan | 21 | + | + | + |
| Iudici *et al*. [11] | 2015 | Retrospective | France | 4 | - | + | + |
| Peco-Antic *et al*. [28] | 2006 | Retrospective | Serbia | 7 | + | + | - |
| Sacri *et al*. [12] | 2015 | Retrospective | France | 38 | - | + | + |
| Sun *et al.* [30] | 2014 | Retrospective | China | 20 | + | + | + |
| Yu *et al*. [29] | 2006 | Retrospective | China | 19 | + | + | + |
|  |  |  |  |  |  |  |  |

**Legend.** + Low risk of bias; - Possible risk of bias; ? Poor reporting

**References**

1. Hunder GG, Arend WP, Bloch DA, et al. The American College of Rheumatology 1990 criteria for the classification of vasculitis. Introduction. Arthritis Rheum 1990;33:1065-7
2. Ozen S, Pistorio A, Iusan SM, et al. EULAR/PRINTO/PRES criteria for Henoch-Schönleinpurpura, childhood polyarteritis nodosa, childhood Wegener granulomatosis and childhood Takayasu arteritis: Ankara 2008. Part II: Final classification criteria. Ann Rheum Dis 2010;69:798-806
3. Jennette JC, Falk RJ, Bacon PA, et al. 2012 revised International Chapel Hill Consensus Conference Nomenclature of Vasculitides. ArthritisRheum 2013;65:1-11
