# Supplementary material for: Childhood-onset granulomatosis with polyangiitis and microscopic polyangiitis: systematic review and meta-analysis
Source: Orphanet J Rare Dis. 2016 Oct 22;11:141. doi: 10.1186/s13023-016-0523-y (PMC5075395; doi:10.1186/s13023-016-0523-y)
Supplement: Additional file 3: — Flow-chart of paper selection. (DOC 61 kb) [file 13023_2016_523_MOESM3_ESM.doc]

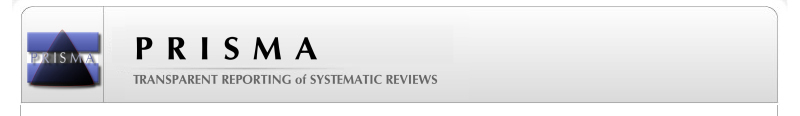
**PRISMA 2009 Flow Diagram**

**Screening**

**Included**

**Eligibility**

**Identification**

Records identified through database searching

(n = 570)

Additional records identified through other sources

(n = 12)

Records after duplicates removed
(n = 582)

Records screened
(n = 582 )

Records excluded (n = 537)

- not pediatric cohorts (n = 213)
- reviews (n = 78)
- case reports (n = 246)

Full-text articles assessed for eligibility
(n = 45)

Full-text articles excluded (n = 23)

- including selected subgroup of AAV patients only (n = 7)

- not reporting AAV baseline patient’s features (n = 16)

Studies included in qualitative synthesis
(n = 22)

Studies included in quantitative synthesis (meta-analysis)
(n = 14 for GPA;

n = 8 for MPA)
