# Supplementary material for: Childhood-onset granulomatosis with polyangiitis and microscopic polyangiitis: systematic review and meta-analysis
Source: Orphanet J Rare Dis. 2016 Oct 22;11:141. doi: 10.1186/s13023-016-0523-y (PMC5075395; doi:10.1186/s13023-016-0523-y)
Supplement: Additional file 4: — Subgroup analysis. (DOCX 21 kb) [file 13023_2016_523_MOESM4_ESM.docx]

**Additional file 4. Subgroup analysis**

**Table S5**. Pooled prevalence of clinical manifestations at presentation in the included studies of childhood-onset granulomatosis with polyangiitis, by speciality and geographic area of origin.

|  | Pooled prevalence (95% CI)  (I^2^) | | | | | | | | | |  |
| --- | --- | --- | --- | --- | --- | --- | --- | --- | --- | --- | --- |
|  | *ENT* | *Systemic* | *Renal* | *Respiratory* | *Joint/muscle* | *Skin* | *Ocular* | *Gastrointestinal* | *Neurologic* | *Cardiovascular* | |
| **All included studies** (*n*=13) | 82 (78-87)  (26) | 73 (55-88)  (88) | 65 (49-79) (84) | 61 (48-74)  (77) | 55 (43-67)  (72) | 44 (32-57)  (75) | 24 (15-34)  (64) | 19 (10-30) (72) | 13 (9-17)  (15) | 4 (1-9)  (61) | |
| **Studies based on paediatric surveys only** (*n*=11) | 83 (77-88)  (26) | 76 (56-92)  (90) | 68 (51-84)  (85) | 62 (46-76)  (80) | 55 (43-67)  (72) | 45 (31-60)  (77) | 22 (12-33)  (69) | 19 (10-30)  (72) | 13 (8-18)  (17) | 4 (0-10)  (69) | |
| **Studies based on non-nephrological surveys only** (*n*=11) | 82 (76-88)  (23) | 70 (48-88) (77) | 60 (42-76)  (85) | 59 (43-74)  (80) | 54 (40-68)  (77) | 44 (30-59)  (78) | 26 (16-37)  (64) | 21 (10-33)  (75) | 17 (10-19)  (2) | 4 (0-10)  (66) | |
| **Studies from Europe** (*n*=7) | 80 (71-88)  (21) | 68 (36-93)  (87) | 67 (41-89)  (79) | 60 (44-75)  (45) | 52 (39-65)  (33) | 55 (32-76)  (72) | 20 (6-38)  (72) | 20 (9-33)  (39) | 7 (2-14)  (0) | 2 (0-5)  (0) | |
| **Studies from North America** *(n*=4) | 83 (76-89)  (0) | 74 (36-99)  (93) | 64 (21-98)  (93) | 67 (34-93)  (89) | 59 (25-89)  (90) | 28 (14-45)  (63) | 26 (9-48)  (78) | 13 (0-37)  (87) | 17 (10-24)  (56) | 9 (0-28)  (87) | |

**Table S6**. Pooled prevalence of clinical manifestations at presentation in the included studies of childhood-onset microscopic polyangiitis, by geographic area of origin

|  | Pooled prevalence (95% CI)  (I^2^) | | | | | | | | | |
| --- | --- | --- | --- | --- | --- | --- | --- | --- | --- | --- |
|  | *Renal* | *Systemic* | *Joint/Muscle* | *Skin* | *Respiratory* | *Gastrointestinal* | *Neurologic* | *Ocular* | *ENT* | *Cardiovascular* |
| **All included studies** (*n*=8) | 94 (89-97)  (47) | 79 (65-90)  (64) | 57 (27-86)  (89) | 44 (27-61)  (73) | 37 (24-52)  (67) | 28 (17-41)  (55) | 18 (7-34)  (72) | 7 (3-11)  (0) | 3 (1-7)  (0) | 2 (0-5)  (0) |
| **Studies from Europe** (*n*=4) | 92 (83-98)  (34) | 91 (78-99)  (34) | 64 (23-97)  (84) | 66 (28-96)  (81) | 30 (15-48)  (31) | 29 (8-56)  (35) | 34 (0-80)  (86) | 6 (1-13)  (0) | 1 (0-5)  (0) | 2 (0-6)  (25) |
| **Studies from Asia** (*n*=4) | 96 (90-99)  (62) | 95 (86-100)  (62) | 65 (9-100)  (64) | 29 (19-40)  (0) | 43 (21-66)  (72) | 29 (16-44)  (44) | 10 (4-18)  (0) | 7 (2-14)  (0) | 6 (1-12)  (20) | 2 (0-6)  (0) |
